# Forest type affects prey foraging of saddleback tamarins, *Saguinus nigrifrons*

**DOI:** 10.1007/s10329-014-0416-4

**Published:** 2014-04-01

**Authors:** Denis Kupsch, Matthias Waltert, Eckhard W. Heymann

**Affiliations:** 1 Behavioral Ecology and Sociobiology Unit, German Primate Center, Göttingen, Germany; 2 Department of Conservation Biology, Georg-August University Göttingen, Bürgerstraße 50, 37073 Göttingen, Germany

**Keywords:** *Saguinus nigrifrons*, Tamarin, Prey foraging, Secondary forest, Primary forest, Habitat use

## Abstract

Callitrichids can persist in secondary forests where they may benefit from elevated prey abundance. However, how tamarins forage for prey in secondary forest compared to primary forest has not been examined. Using scan and focal sampling, we compared prey foraging and capture success of two groups of *Saguinus nigrifrons* in north-eastern Peru: one ranging in primary forest, the other with access to a 10-year-old anthropogenic secondary forest. There was a trend for more prey search in the secondary forest, but prey feeding, capture success and size were lower compared to the primary forest. Tamarins avoided the forest floor, used vertical supports less often and searched on a lower variety of substrates in the secondary forest. In the secondary forest, tamarins did not capture flushed prey, which make up a substantial part of the total prey captures biomass in primary forests. Reduced prey capture success is unlikely to reflect reduced prey availability, since more Orthoptera were found in secondary forest through ultrasonic surveys. Therefore, the prey search activity of *S. nigrifrons* in young secondary forests seemed rather opportunistic, presumably influenced by altered predation patterns, vegetation structure, as well as prey diversity.

**Electronic supplementary material:**

The online version of this article (doi:10.1007/s10329-014-0416-4) contains supplementary material, which is available to authorized users.

## Introduction

Human activities increasingly affect natural forest ecosystems of the tropics (Wright and Muller-Landau 2006; FAO 2007). In particular, timber harvesting and agriculture push back primary rain forests, and land abandonment causes the development of secondary forest ecosystems (Wright 2005), which differ in structure and species composition (Johns 1997; Laurance 2007). As secondary forests become more prevalent in the tropics, it is of increasing importance to explore their inherent ecosystem processes as well as community compositions and whether they meet the ecological importance of primary forests (Daily 2001; Vandermeer and Perfecto 2007; Liebsch et al. 2008). This is especially true in the light of the current biodiversity crisis, which is primarily linked to habitat conversion (Pimm et al. 1995; Laurance and Wright 2009). Studies on alterations of biodiversity resulting from anthropogenic influence have typically focused on invertebrates (e.g. Floren and Linsenmair 2005; Barlow et al. 2007a; Gardner et al. 2008). However, if we are to understand the ecology and conservation value of secondary forests, their suitability for vertebrates also needs to be assessed (e.g. Gray et al. 2007; Barlow et al. 2007b).

Most tamarins, genus *Saguinus,* are distributed throughout the Amazon Basin and have been variably considered as habitat generalists, preferring a mix of forest types (Garber 1993), or specialists preferring secondary and successional forest and edge habitat (Rylands 1996). A higher abundance of invertebrate prey — which represents a substantial component of tamarin diets besides exudates and fruits (Peres 1993; Nickle and Heymann 1996) — in secondary and edge forest is supposed to lead tamarins to prefer such forest types (Bernstein et al. 1976; Terborgh 1983; Yoneda 1984a; Schwarzkopf and Rylands 1989; Rylands 1996). Since tamarins may promote natural forest regeneration of secondary forests as important seed dispersers (Culot et al. 2010), it is essential to study whether, and how, tamarins utilize this forest type. However, prey foraging and capture rates of tamarins have never been compared between primary and secondary forest or forest edge.

Based on the assumption that tamarins benefit from utilization of secondary forest, we hypothesised that prey foraging and capture rates would be higher in secondary forest than in primary forest. For this, we compared activity budgets, diet compositions, prey foraging strategies, capture techniques and rates of a group of saddleback tamarins, *Saguinus nigrifrons* — a recent revision has elevated *Saguinus fuscicollis nigrifrons* to species rank (Matauschek et al. 2011) — living in primary forest with a group that had access to a 10-year-old secondary forest in Amazonian Peru.

## Methods

### Study site

The study was carried out at the Estación Biológica Quebrada Blanco (EBQB) between March 14 and June 27, 2011. The EBQB is located in north-eastern Peru (4°21′S, 73°09′W) on the right bank of Quebrada Blanco, a white-water affluent of the Rio Tahuayo, at an altitude of 110-140 m asl. The majority of the study area is primary terra firme forest with embedded swampy sections. The southern study area includes a secondary forest originating from former agricultural use (first for crop cultivation, later as buffalo pasture). Since 2001 the pasture has remained unused and regenerates, with typical pioneer plants like *Cecropia* sp. dominating the tree layer.

In June 2011 we surveyed the vegetation structure in nine randomly selected points in each of the two habitats. Vegetation cover — estimated visually within a radius of 10 m around the sampling point — was higher in primary than in secondary forest in all forest strata above 5 m height (Online Resource Fig. S1a). Estimated epiphyte coverage was 2.9 ± 2.9 % (mean ± SD) in primary and 0.9 ± 1.0 % in secondary forest, dominated by Araceae and Bromeliaceae (Wörner 2007). Other structure parameters were recorded with angle count-sampling (Bitterlich 1952, Kramer and Akça 2008) using Kramer’s dendrometer under a basal area factor of *k* = 2. We subtracted the influence of inclination and re-measured trunks at the threshold. Primary and secondary forest composition consisted of 86.8 and 92.1 % trees, 4.4 and 1.4 % palms, 0.7 and 0.0 % lianas, as well as 8.1 and 6.5 % deadwood, respectively (Online Resource Fig. S1b). While the secondary forest is dominated by trees with *dbh* < 15 cm (diameter at breast-height), tree size composition in primary forest showed a relatively even distribution (Online Resource Fig. S1c). In addition, we calculated mean heights of 24.5 ± 3.1 and 18.1 ± 2.7 m, total basal area of 29 m^2^/ha and 16.7 m^2^/ha, and stand density (for trees with *dbh* > 5 cm) of 2,495 trees/ha and 2,443 trees/ha for primary and secondary forest, respectively.

Mean annual rainfall in 2011 was about 2,300 mm (measured at Tamshiyacu, 40.4 km north of the EBQB, data provided by the Servicio Nacional de Meteorologia e Hidrologia del Peru). Our study period corresponded to the wet season, with maxima in April (269 mm) and May (368 mm), and the transition between late wet season and beginning of a drier season starting in June (181 mm).

### Study groups

We observed two groups of *S. nigrifrons* in blocks of 6 subsequent days in a regular weekly change. Group 1 (3 adults, 1 subadult and 2 infants born in February) had access to secondary forest. Group 3 (2–3 adults, one of which died in April, 2 subadults and 2 infants born in May) lived directly north of group 1 solely in primary forest and served as a reference group to control for group-specific patterns when comparing foraging behaviour of group 1 in primary and secondary forest.

Observation usually started from 0600 hours, when the tamarins left a sleeping site, and continued until the afternoon at about 1600 hours, when they entered another sleeping site. In total, group 1 was observed for 202 h over 28 days and group 3 for 191 h over 29 days. Both groups formed stable mixed-species troops with moustached tamarins *Saguinus mystax* and were well habituated to the presence of human observers.

### Observational methods

We used three different methods to collect behavioural data from the study groups, excluding infants. Instantaneous scan-sampling (Martin and Bateson 2007) at 15-min intervals focused on activity budget and diet composition. We recorded the type of activity (locomotion, resting, social interaction, prey searching, fruit feeding, exudate feeding, prey feeding, other) of each tamarin that became visible within 30 s after the scan sampling-point indicated by the beep of a timer. We obtained 745 activity records for group 1 (681 in primary, 64 in secondary forest), and 655 for group 3. At each scan-sample point, we recorded the group position using a Garmin GPSMap 76CSx. Between scan-sampling points, focal sampling with continuous recording (Martin and Bateson 2007) was employed to record prey searching strategies, capture techniques, prey characteristics and capture rates. The length of focal samples was 10 min, during which the focal individual had to be visible for at least 5 min. The selection of focal individuals followed a previously set rotational scheme. If we could not find the individual at the top of the scheme within 2 min, we selected the next individual. If this could not be found either, we started a new search again with the previous individual. By using this procedure we ensured an even distribution of the number of focal protocols over all studied individuals. We recorded 66.7 h of focal sampling for group 1 (61.0 h in primary and 5.7 h in secondary forest) and 55.7 h for group 3. During focal sampling the same activities as in scan sampling were registered. Additionally, we recorded support type and orientation as well as substrate type and height during prey search. We also categorized the technique of each successful prey capture: direct capture from open microhabitats, low intensity manipulation, e.g. opening epiphytes or unrolling dry leaves, and high intensity manipulation including breaking or biting open substrates (see also Nadjafzadeh and Heymann 2008). If captured directly, we registered whether this arose from prey flushing, meaning events where prey items fled from other tamarins. Also, we recorded colour and size of prey items. We defined prey capture success (*S*_*i*_) as the rate of captures per prey search time in focal samples. Outside of focal and scan sampling, other prey feeding events were recorded noting group, forest type, date, time and prey type, with as much detail as possible. We collected prey items discarded by the tamarins, e.g. orthopteran tegmina and hind wings, for later identification. To increase interobserver reliability, especially on height and in situ prey size estimation, we carried out a multi-week tutorial with the field assistants prior to data collection.

### Prey abundance

We concentrated our survey of potential prey abundances in primary and secondary forest on nocturnal katydids since this is the dominant part of the tamarins’ prey (Nickle and Heymann 1996; Smith 2000; Nadjafzadeh and Heymann 2008). Katydids belong to the family Tettigoniidae, order Orthoptera, and produce distinct species-specific stridulation sounds. Therefore, we recorded prey abundance as the number of singing orthopteran individuals on three randomly placed 50-m transects in primary and secondary forest, respectively. All transects were walked with the same velocity within 10 min using a Petersson D200 heterodyne ultrasonic detector during rainless nights. To account for species-specific activity patterns (Belwood 1990; Nickle and Heymann 1996) we repeated the survey at three different times (1900, 2300 and 0300 hours) once a month from March to June.

### Data analyses

We carried out statistical analyses with R 2.12.1 (R Development Core Team 2010) using the packages stats, vegan (Oksanen et al. 2011), and lme4 (Bates et al. 2011). For the analyses of activity budgets and diet composition, only complete observation days were used. We used Fisher’s exact test to compare activity budgets, diet composition, prey searching strategies and capture techniques between groups and habitat types. The function *fisher.test* in R uses a subroutine (FEXACT) to execute Fisher’s exact tests on contingency tables larger than 2 × 2 (Mehta and Patel 1986; Clarkson et al. 1993). All tests were two-tailed at a significance level of *α* < 0.05. For each test where the null hypothesis had to be rejected, we performed additional multiple testing to obtain specific information about diverging categories. We used the Bonferroni correction to correct the significance level in multiple tests.

Due to unequal numbers and variances in the comparative analysis of prey size, we employed Welch’s unequal variance *t*-test with previously ranked values instead of Mann–Whitney *U*-test (Ruxton 2006).

We analysed the diurnal distribution of secondary forest utilization of group 1 and compared it with diurnal patterns in fruit feeding and prey search.

We calculated the overlap of captured prey items between groups based on Morisita’s unmodified index of similarity without log-transforming data (Krebs 1999):

$$C = \frac{{2\sum {p_{ij} } p_{ik} }}{{\sum\nolimits_{{}}^{n} {p_{ij} \left[ {\left( {n_{ij} - 1} \right)/\left( {N_{j} - 1} \right)} \right] + \sum\nolimits_{{}}^{n} {p_{ik} \left[ {\left( {n_{ik} - 1} \right)/\left( {N_{k} - 1} \right)} \right]} } }},$$

where *C* is Morisita’s index, *p*_*ij*_ and *p*_*ik*_ are the proportions of prey item *i* in the total prey used by groups 1 and 3, respectively, *n*_*ij*_ and *n*_*ik*_ are the numbers of individuals that use prey item *i* in groups 1 and 3, respectively, and *N*_*i*_ and *N*_*k*_ are the total numbers of individuals in each group. This index produces a minimum bias of abundance and diversity in different data sets (Wolda 1981; Smith and Zaret 1982).

For taxonomic identification of collected prey items, we used literature (Beier 1962; Belwood 1990; Nickle and Castner 1995; Nickle and Heymann 1996; Bartlett and Bartlett 2003), the species online files for Orthoptera (http://orthoptera.speciesfile.org) and Phasmida (http://phasmida.speciesfile.org), a reference collection of tamarin prey provided by Andrew C. Smith (see also Smith 2000) as well as assistance by experts for Orthoptera, Holger Braun (División Entomología, Museo de La Plata, Argentina), and Phasmida, Sven Bradler (Johann-Friedrich-Blumenbach Institute of Zoology, Göttingen, Germany). Where exact taxonomic identification was not viable due to insufficient prey remains, the items were classified into morphotypes using colour and size as categories.

We assessed prey abundance using the generalized linear mixed model function *lmer* (Bates et al. 2011) with a significance level at *p* < 0.05. We set night-time as random, forest type and month as fixed effects.

GPS positions were processed in ESRI ArcGIS 9.3. We performed fixed kernel home-range estimation following Worton (1989) with the software extension ‘Home-Range-Tools’ (Rodgers et al. 2007). To analyse habitat utilization, we calculated intensities of primary and secondary forest habitat use indices (*H*_*i*_) for group 1, basically following Neu et al. (1974):

$$H_{i} = \log \frac{{\text{freq}_{\text{obs}} }}{{\text{freq}_{\exp } }}$$

This method compares the frequencies between observed and expected values for each habitat type. Observed frequencies were obtained from recorded GPS positions. A buffering of these points by 5 m represents the distribution of the tamarin group in the field and converts the point data to polygon. Krebs (1999) suggested using habitat type availability as expected frequencies. Thus, we calculated the proportions of forest types within the home range of group 1 based on the 100 % minimum convex polygon (MCP) (Mohr 1947), which we also buffered by 5 m. We log-transformed the term to get an index value between −1 (avoidance) and +1 (preference). Differences between expected and observed frequencies were tested using Fisher’s exact test.

All presented statistical information were referred to Fisher’s exact test, except where otherwise stated.

## Results

### Home-range and habitat use

**Fig. 1:**
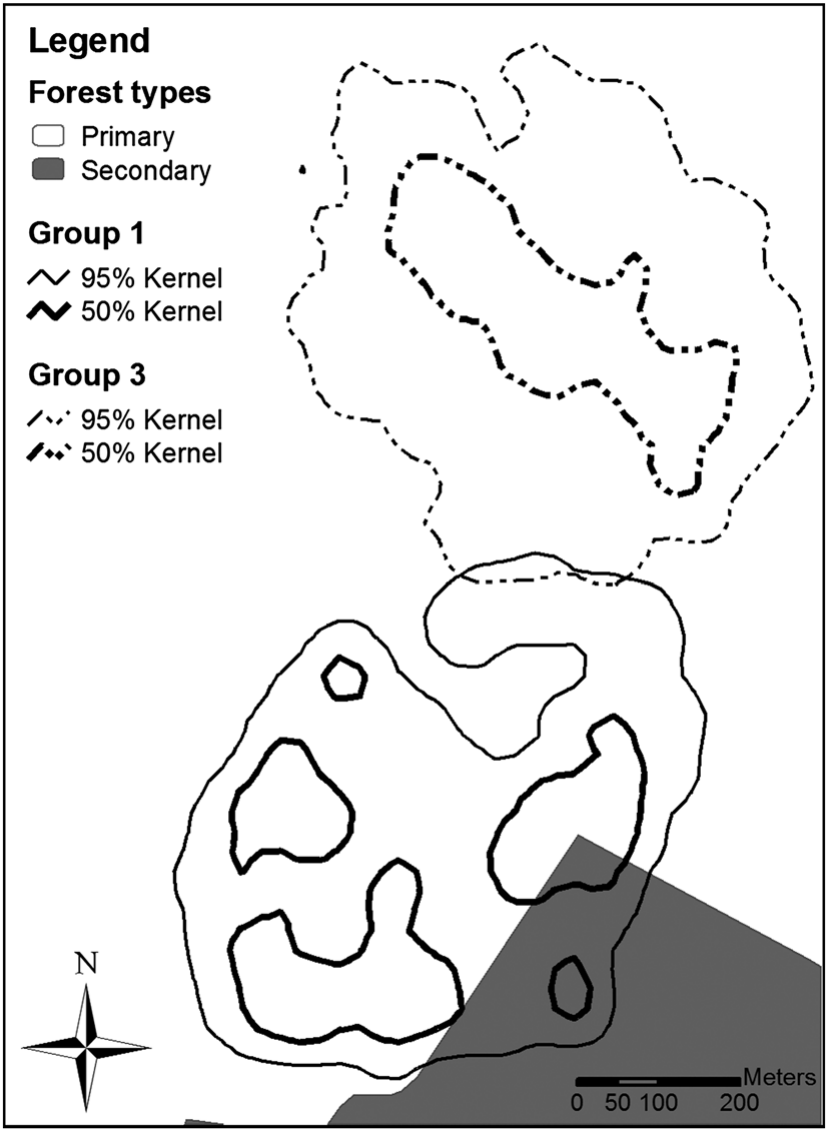
Study area and kernel home ranges of groups 1 and 3 (smoothing factor *h* = 25)

Home-range size (95 % kernel, based on 740 GPS positions) was 28.6 ha for group 1 and 29.0 ha for group 3 (619 GPS positions); core areas (50 % kernel) were 8.0 and 6.7 ha, respectively (Fig. 1).

**Fig. 2:**
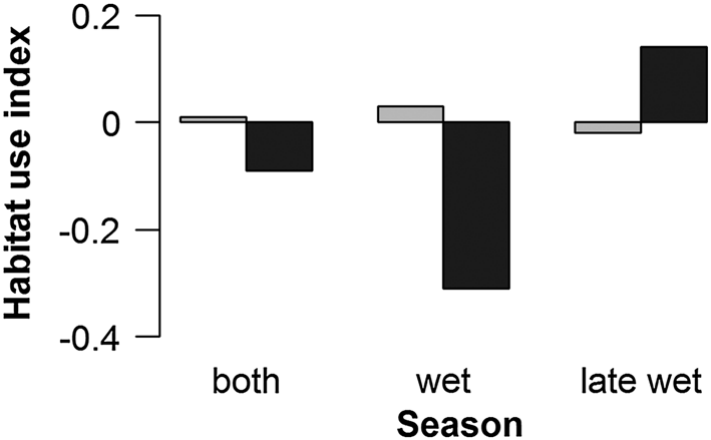
Habitat use indices of group 1 for primary *(light grey*) and secondary forest (*dark grey*) in different seasons

Over the entire study period, group 1 showed no clear preference or avoidance of primary or secondary forest (Fig. 2). However, comparing the seasonal utilization rates, we detected a significant increase of secondary forest use in the late wet season (*p* = 0.040).

### Activity budget and diet composition

Groups 1 and 3 did not differ in activity budgets (*p* = 0.723; Online Resource Fig. S2). There was also no difference between group 1 in primary forest only and group 3 (*p* = 0.728), and between group 1 in primary vs secondary forest (*p* = 0.466). Time allocated to prey search tended to be higher in secondary forest but the difference was not significant.

**Fig. 3:**
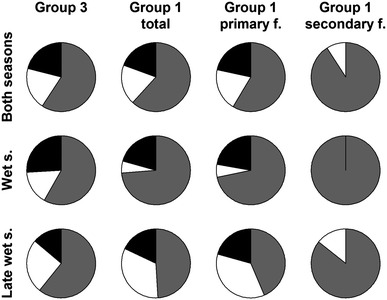
Diet composition of both groups in different forest types and seasons; *grey*: fruit, *white*: exudate, *black*: prey; data based on scan sampling

Diet composition of group 1 varied significantly between forest types (*p* < 0.001) with more fruit (*p* < 0.001) and less exudate feeding (*p* = 0.043) in secondary forest across both seasons (Fig. 3). No prey feeding was recorded in secondary forest during scan sampling. Compared to the wet season, group 1 consumed fewer fruits (*p* < 0.001) and more exudates (*p* < 0.001) in the late wet season.

### Prey searching strategies

**Fig. 4:**
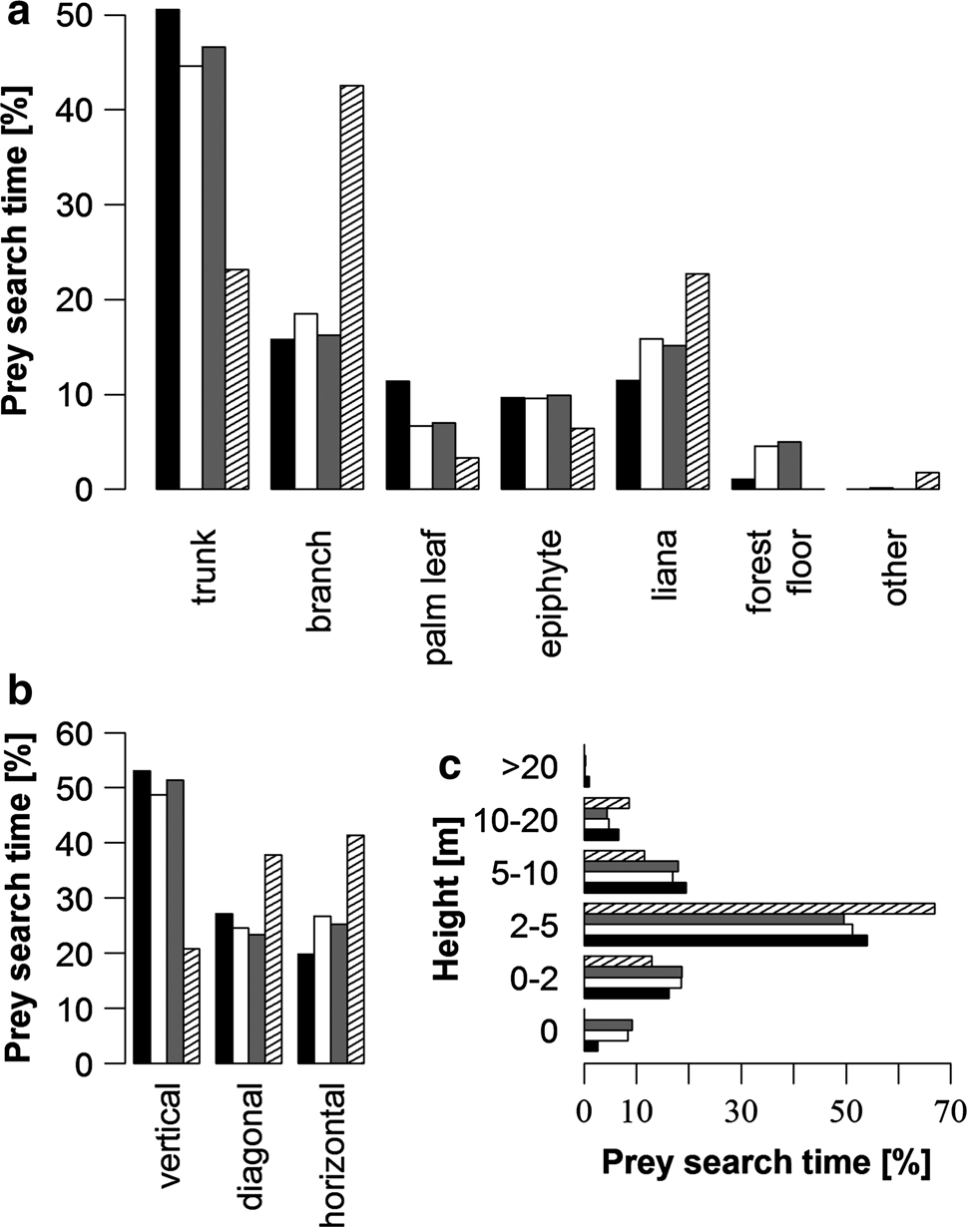
**a** type of support, **b** support orientation and **c** height used for prey foraging in group 3 (*black*), group 1 (*white*), group 1 in primary (*grey*) and secondary forest (*striped*) only

**Fig. 5:**
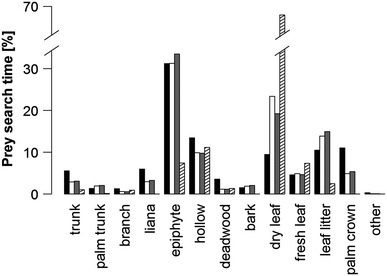
Substrates searched for prey in group 3 (*black*), group 1 (*white*), group 1 in primary (*grey*) and secondary forest (*striped*) only

Prey search strategies did not differ (Figs. 4, 5) between groups 1 and 3 (*p* = n.s. in all categories; prey search time group 1: 541 min; group 3: 315 min) or group 1 in primary forest only (*p* = n.s. in all categories; prey search time group 1_prim_: 495 min). Prey searching in group 1 varied significantly between secondary and primary forest in support (*p* < 0.001; prey search time group 1_sec_: 46 min), orientation (*p* < 0.001), substrate (*p* < 0.001) and height (*p* = 0.020). In the secondary forest trunks were used less (*p* < 0.001) and branches more intensively (*p* < 0.001) as a support, but never the forest floor. Consequently, orientation of support was less often vertical (*p* < 0.001) in the secondary forest. In the secondary forest, group 1 searched more intensively in dry leaves (*p* < 0.001) and less in epiphytes (*p* < 0.001) and leaf litter (*p* = 0.005) than in the primary forest.

### Prey capture techniques

Prey capture techniques and prey characterization did not differ between groups 3 and 1 (*p* = n.s. in all categories; *n*_1_ = 91; *n*_3_ = 42 capture events; see also Online Resource Fig. S3) or group 1 in primary forest only (*p* = n.s.; *n*_1prim_ = 86). Due to a small number of prey captures during focal sampling in the secondary forest (*n*_1sec_ = 5), we have not conducted statistical comparisons. However, high intensity manipulation, prey flushing events and captures of green prey items were not observed in secondary forest.

Prey items captured by group 3 were significantly larger than those captured by group 1 in total (*x*_1_ = 3.6 ± 2.7 cm; *x*_3_ = 4.7 ± 3.4 cm; Welch’s *t*-test: *t* = 2.79; *df* = 181.04; *p* = 0.004; *n*_1_ = 177; *n*_3_ = 99) and for the portion of primary forest (*x*_1prim_ = 3.7 ± 2.8 cm; Welch’s *t*-test: *t* = 3.37; *df* = 179.16; *p* = 0.011; *n*_1prim_ = 163). Moreover, prey captured by group 1 in secondary forest was significantly smaller than in primary forest (*x*_1sec_ = 2.4 ± 1.0 cm; Welch’s *t*-test: *t* = 12.04; *df* = 62.78; *p* < 0.001; *n*_1sec_ = 14). In total, captured prey was almost twice as big when it arose from prey flushing (*x*_flushed_ = 5.0 ± 3.3 cm; *x*_notflushed_ = 2.9 ± 2.2 cm; Welch’s *t*-test: *t* = 4.12; *df* = 40.51; *p* < 0.001; *n*_flushed_ = 24; *n*_notflushed_ = 79).

Data shown in Table 1 were obtained from the type of substrate where a prey item was captured. The types of microhabitat group 3 searched prior to captures differed significantly from those of group 1 (*p* < 0.001) and group 1 in primary forest (*p* = 0.002). Due to insufficient numbers in the categories to test, we have not conducted further statistical analysis. However, both groups reached relatively high capture rates in leaf litter, epiphytes and hollows, such as knotholes.

**Table 1:** Substrates of prey items captured by group 1 (primary and secondary forest) and group 3 during focal sampling

	Group 3		Group 1		Group 1, primary forest		Group 1, secondary forest	
	No.	%	No.	%	No.	%	No.	%
Trunk	5	11.9	10	11.0	9	10.5	1	20.0
Palm trunk	–	–	–	–	–	–	–	–
Branch	1	2.4	–	–	–	–	–	–
Liana	1	2.4	1	1.1	1	1.2	–	–
Epiphyte	6	14.3	16	17.6	15	17.4	1	20.0
Hollow	12	28.6	10	11.0	10	11.6	–	–
Deadwood	1	2.4	–	–	–	–	–	–
Bark	2	4.8	1	1.1	1	1.2	–	–
Dry leaf	2	4.8	14	15.4	13	15.1	1	20.0
Fresh leaf	1	2.4	10	11.0	8	9.3	2	40.0
Leaf litter	11	26.2	28	30.8	28	32.6	–	–
Palm canopy	–	–	1	1.1	1	1.2	–	–
Other	–	–	–	–	–	–	–	–
Total	42	100	91	100	86	100	5	100

### Diurnal variation of habitat use and foraging behaviour

**Fig. 6:**
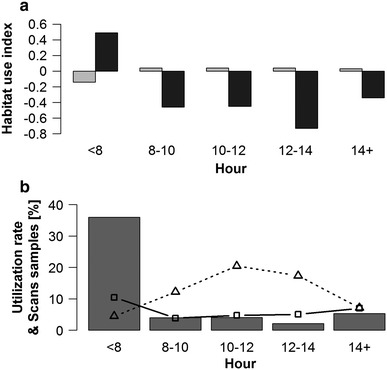
Diurnal variation of **a** habitat use of primary (*grey*) and secondary forest (*black*); **b** secondary forest utilization (*bars*), prey search (*triangles* and *dotted line*) and fruit feeding (*squares* and *solid line*) activity in group 1

Group 1 showed preference for secondary forest in the first 2 h of the day (Fig. 6a; *H*_sec_ = 0.49; *H*_prim_ = −0.14; *p* < 0.001; *n* = 129 GPS positions) and avoidance during the rest of the day (significant for 1200–1400 hours: *H*_sec_ = −0.73; *H*_prim_ = 0.04; *p* = 0.010; *n* = 156 GPS positions). The distribution of fruit feeding in group 1 over the day went in parallel with secondary forest utilization, but prey search peaked between 1000 and 1400 hours when secondary forest use was low (Fig. 6b). The diurnal distribution of fruit feeding and prey search did not differ between groups 1 and 3 (fruit: *p* = 0.982; prey search: *p* = 0.588), thus, group-specific idiosyncrasies for patterns observed in group 1 can be excluded.

Because habitat utilization in group 1 showed a diurnal variation, we compared the activity budget and prey search strategies in group 1 of the periods 0600–0800 and 0800–1600 hours. To control for forest type, we excluded secondary forest data from the analysis. In the early morning hours the tamarins travelled more (*p* = 0.003; *n*_bef8_ = 89; *n*_aft8_ = 592 activity records) but showed less prey searching activity (*p* < 0.001) than during the rest of the day. Prey searching varied significantly during the daytime in substrates (*p* = 0.04; prey search time before 0800 hours: 29 min; after 0800 hours: 466 min) but not in support, orientation and height. We did not test for diurnal differences in prey capture techniques due to insufficient sample sizes in the early morning hours (*n*_bef8_ = 6).

### Prey spectrum

Orthopterans were the most dominant prey items (Online Resource Table S1), independent of group (group 1: 74 %; group 3: 77 %) or forest type (secondary forest: 69 %; primary forest: 74 %). Additionally, both groups consumed small portions of Araneida, Scorpionida, Vertebrata and Phasmatodea. Only group 3 captured Coleoptera, whereas group 1 uniquely fed on Blattodea, Mantodea and eggs of Araneida. The tettigoniid *Typophyllum mortuifolium* was only recorded in secondary forest. A Morisita Index of 0.852 (excluding unidentified prey: 0.763) indicates a high overlap of prey items comparing group 3 and the portion of primary forest in group 1. This value decreases slightly to 0.816 (excluding unidentified prey: 0.727) when adding the prey items of secondary forest. Both comparisons show significant differences (both *p* < 0.001; excluding unidentified prey: both *p* < 0.001). Prey spectrum did not differ between secondary and primary forest (*p* = 0.836; excluding unidentified prey: *p* = 0.965).

### Prey capture rate

Group 1 was more successful in prey capturing than group 3 (*S*_1_ = 10.1 ind/h_preysearch_; *S*_3_ = 8.0 ind/h_preysearch_). In primary forest the prey capture rate of group 1 was higher (*S*_1prim_ = 10.4 ind/h_preysearch_) than in secondary forest (*S*_1sec_ = 6.5 ind/h_preysearch_).

### Prey abundance

The number of calling Orthoptera was significantly higher in secondary than in primary forest (*N*_prim_ = 11.2 ± 2.5; * N*_sec_ = 15.1 ± 4.4; *lmer*: *z* = 34.53; *p* < 0.001). There was a significant influence of month, with highest numbers in June (*N*_June_ = 16.5 ± 5.0; *lmer*: *z* = 33.52; *p* < 0.001). We excluded the interaction term between forest type and month from the final model due to a non-significant influence.

## Discussion

### Utilization of forest types

In contrast to other studies on the ranging behaviour of the genus *Saguinus* (Bernstein et al. 1976; Yoneda 1984a; Schwarzkopf and Rylands 1989; Vulinec et al. 2006), we could not detect a general preference of secondary forest by group 1. These differences could mainly reflect a variable definition of the term ‘secondary forest’ with a wide range of age classes and vegetation types from which the studied forest developed. Clear-cutting, for instance, generally eliminates most species from a site and destroys soil structure and nutrient capital, while selective logging and similar human interferences maintain these structural parameters to a greater or lesser extent (Corlett 1994). In turn, a former use of land as plantation can lead to influences on species composition through introduced crops (Chokkalingam and De Jong 2001). Thus, starting conditions, such as the former type of vegetation or anthropogenic exploitation, influence the succession and recovery rate. These patterns are also linked to the age of the secondary forest, which highly affects productivity rates and material flows within the forest system (Brown and Lugo 1990). Comparisons between studies in regard to the intricacy of the term ‘secondary forests’ should therefore be made with caution. Among the previously listed studies, only Bernstein et al. (1976) worked in a comparable secondary forest younger than 10 years that emerged on a clear-felled area, but their findings on habitat utilization of tamarins were supported by only six sightings. Indeed, for *S. nigrifrons,* Culot (2009) showed that during the early stages of forest succession, the frequencies of visits by tamarins increased gradually.

Our results revealed marked differences in the prey foraging behaviour of *S. nigrifrons* between primary and secondary forest. However, these do not seem to be due to group-specific characteristics of group 1, because its activity budget and diet composition did not differ noticeably compared to group 3.

Although prey was not represented in the diet composition in secondary forest, we recorded prolonged prey searching activities, which is in line with other studies (Bernstein et al. 1976; Terborgh 1983; Yoneda 1984a; Schwarzkopf and Rylands 1989; Rylands 1996). This might be due to less (albeit not significantly) frequent resting and social activities, which were mainly reduced to stops during heavy rain and scent-marking behaviour close to the home-range boundaries, indicating predation-sensitive behaviour (Garber and Bicca-Marques 2002). Indeed, the structural deficiency in secondary forest, especially the lack of a closed canopy, increases the risk of predation by raptors (Vidal and Cintra 2006; Oliveira and Dietz 2011), which is a substantial threat to tamarins (Terborgh 1983; Heymann 1990b).

The topographical position might also have influenced prey foraging intensity. The secondary forest was located along the home-range boundary of group 1. Peres (1992a) found that saddleback tamarins spend more time searching for prey close to the boundaries of their range, presumably to minimize food loss due to prey foraging by neighbouring groups in overlap areas of the home-range. This investment in time and energy is a result of territorial defence and, therefore, independent of the actual quality of the foraging site.

In accordance with Lopes and Ferrari (1994), we observed that the tamarins frequently used the periphery of abundant fruit sources for prey search activities. Regularly the individuals of *S. nigrifrons* had to wait for their congeners to finish feeding because *S. mystax* holds the dominant position within the mixed-species troop, allowing the first access to fruiting trees (Heymann 1990a; Peres 1992a). Besides, saddlebacks used the movements to and away from the fruit source for prey search (‘travel foraging’) instead of fast locomotion. Although the visits to secondary forest were not motivated by the prospect of prey search, the behaviour of the tamarins actually led to an increase of prey search activity within the time budget.

The seasonal variation in the utilization of the secondary forest is in line with the findings of Culot (2009) at the same study site during the same seasons. Culot (2009) argued that the secondary forest provides fruit sources in times of fruit scarcity. Indeed, in June fruit feeding slightly increased in the secondary forest, and especially during the first two hours of the day it was used as a preferred fruit foraging area. Smith (2000) argued that due to low energy levels at the beginning of the day, tamarins urgently need a source of fast energy. Thus, tamarins usually visit abundant fruit sources, which become rare in the dry season (Lopes and Ferrari 1994; Peres 1994; Culot 2009). Although the tamarins always slept outside of the secondary forest, in June they headed for one such tree every morning directly after rising, which indicates the importance of abundant fruit sources in times of fruit scarcity.

That the tamarins mainly visited secondary forest during the morning hours may also be a result of microclimatic changes during the day. While the closed canopy in primary forest provides an effective protection against direct solar radiation, the more open vegetation in secondary forest increases the risk of hyperthermia, especially in the drier months with fewer clouds, and during midday and early afternoon (Hill 2005).

On the one hand, the strong diurnal variation of secondary forest use illustrates the selective utilization of that habitat type by *S. nigrifrons*. On the other hand, it may also influence the patterns found in prey foraging behaviour within the secondary forest. However, even though we may not exclude time of day as a possible influence on saddleback prey foraging, there is also no indication for such an effect, neither in our data nor in the literature.

Our results contradict the assumption that saddleback tamarins benefit from increased prey abundances because of higher proportions of fresh leaves during the early regrowth stages of secondary forests (Schwarzkopf and Rylands 1989; Peres 1992b; Rylands 1996). Although we recorded higher densities of Orthoptera in secondary forest, the tamarins fed on these much less than in primary forest. On the one hand, this could be due to methodical reasons: the thicker foliage of primary forests can obstruct the oscillations of ultrasounds, such as orthopteran stridulations (Diwakar and Balakrishnan 2007). This is especially true for the Pseudophyllinae that belong to the preferred prey of *S. nigrifrons* (Peres 1993; Smith 2000; Online Resource Table S1) and often perform high-frequency stridulation (Montealegre et al. 2006). On the other hand, however, this may also be caused by varying species compositions between forest types. Using arboreal ants as model organisms, Floren and Linsenmair (2005) showed that in tropical secondary forests usually only a few species become dominant. Barlow et al. (2007a) demonstrated for grasshoppers (Orthoptera; Acridiidae) that <40 % of primary forest species occurred in secondary forest and <10 % of species were unique to it, which is mainly due to higher temperatures (Ruiz-Guerra et al. 2012) or missing microhabitats serving as diurnal shelters, like palms or epiphytes (Belwood 1990). Thus it is likely that, although the overall prey abundance increases in secondary forest, the potential prey diversity decreases. As we know that *S. nigrifrons* consumes only a fraction of the more than 300 orthopteran species (Nickle and Heymann 1996; Smith 2000) native to north-eastern Peru (Nickle and Castner 1995), the restricted food resource of orthopteran prey (Peres 1993; Nickle and Heymann 1996) seems to be even more limited in secondary forest.

### Patterns of prey foraging influenced by forest type

Our findings on prey search strategies and capture techniques in both groups are in accordance with other studies (e.g. Terborgh 1983; Peres 1993; Nadjafzadeh and Heymann 2008). Thus, in general, *S. nigrifrons* is a highly manipulative forager of the lower forest strata, using a wide range of different support types and substrates. Relatively long slender hand shapes enable saddleback tamarins to exploit concealed microhabitats, like epiphytes and knotholes (Garber 1991; Bicca-Marques 1999), mainly for large prey items, while elongated fore- and hindlimbs facilitate movement and foraging in the lower forest strata through ‘clinging and leaping’ from trunk to trunk (Garber and Leigh 2001). We found no differences in prey search strategies and capture techniques between group 3 and group 1 as well as group 1 in primary forest only, but great differences between forest types.

In secondary forest we never observed tamarins on the forest floor. The avoidance of this microhabitat could be one reason for the low capture rate in secondary forest since most prey, especially concealed Orthoptera, jump to the ground to hide in leaf litter when detected by predators (Peres 1992b; Nickle and Castner 1995). In contrast, group 1 obtained nearly one third of the captured prey items from the forest floor in primary forest. Besides open canopy, a dense understorey, as found in secondary forest, raises the predation risk in tamarin groups as a consequence of poor visibility of predators, such as felids and snakes (Vidal and Cintra 2006). Since prey flushing is a prevailing benefit for *S. nigrifrons* in mixed-species troops (Peres 1992b), a general absence of captures of flushed prey would be noteworthy. In a study on the same tamarin species combination, flushed prey made up for more than 40 % of captured items and 70 % of captured biomass of saddlebacks (Peres 1992b). This is also in line with the larger size of flushed prey we found, and highlights its importance for the protein uptake of saddlebacks.

The diversity of substrates utilized by *S. nigrifrons* for prey search markedly decreased in the poorly heterogeneous vegetation of the secondary forest. Generally, *S. nigrifrons* intensively exploits bromeliads like *Guzmania vittata* and *G. lingulata* for prey foraging (Peres 1993; this study). However, epiphytes and palms, as well as lichen and mosses, which are important for many Pseudophyllinae to conceal themselves during the day (Belwood 1990; Nickle and Heymann 1996), need comparatively more time to establish than pioneer vegetation and are thus sparsely distributed in young regrowth forests (Costa 1999; Wörner 2007; Online Resource Fig. S1). As a result, the saddlebacks almost exclusively — albeit not successfully — explored dried curled leaves for prey, which are abundant in secondary forest due to a dominance of the pioneer tree *Cecropia* sp. that produces large short-lived leaves (Clark and Clark 1992).

In general, prey capture success was much lower in secondary than in primary forest. Assuming that prey accessibility determines capture success (Terborgh 1983), the secondary forest appears to be an unsuitable habitat for prey foraging. In addition, *S. nigrifrons* mostly captured prey items of small size, mainly orthopteran instars, Grylloidae and Arachnida, in secondary forest, although it is the most specialized tamarin species in regard to large prey (Yoneda 1984b; Peres 1992b; Nickle and Heymann 1996). Moreover, Proscopiidae, which account for more than 17 % of the total number of ortopteran prey items, and achieve body lengths up to 20 cm, were not part of the prey spectrum in the secondary forest. The lack of this prey species group alone may lead to a major decline of protein uptake in secondary forest.

## Conclusions

Our results represent one of the first attempts to evaluate the utilization of young secondary forest by *Saguinus nigrifrons* under the perspective of prey foraging. We did not find a general preference of secondary forest by saddleback tamarins. Moreover, their main motivation in visiting the secondary forest seems to be the use of abundant fruit sources. Although the time tamarins spend on prey searching is relatively longer in secondary forest, other important parameters are contrasting: no recorded prey feeding during scan sampling, low prey capture success and smaller prey sizes. Thus, we consider *S. nigrifrons* to be an opportunistic prey forager in secondary forest. This pattern is interpreted as a result of higher predation risk and poorer vegetation structure. The same factors influence the methods of prey foraging. Prey search in secondary forest is mainly reduced to exploration of dried curled leaves, while capture events emerging from prey flushing seem not to occur. In addition, the diversity of the prey spectrum and prey size both decrease significantly.

In summary, young secondary forest does not seem to serve as a suitable prey foraging habitat for *S. nigrifrons*, although this species is considered to be highly flexible in habitat utilization (Rylands 1996). It remains an open question whether this also applies for other types and ages of secondary forest. Further comparable studies in this context could also enrich the discussion on the conservation value of secondary forests (Dent and Wright 2009; Waltert et al. 2011) because this methodical approach does not (just) focus on species number and abundance as indicators for biodiversity, but rather on performances of ecological demands of species.

## Electronic supplementary material

Below is the link to the electronic supplementary material.

Supplementary material 1 (PDF 1,152 kb)
