# Supplementary material for: Forest type affects prey foraging of saddleback tamarins, Saguinus nigrifrons
Source: Primates. 2014 Apr 1;55(3):403–13. doi: 10.1007/s10329-014-0416-4 (PMC4082136; doi:10.1007/s10329-014-0416-4)
Supplement: Supplementary file 1 — Supplementary material 1 (PDF 1,152 kb) [file 10329_2014_416_MOESM1_ESM.pdf]

Supplementary Material – Online Resource

Forest type affects prey foraging of saddleback tamarins *Saguinus nigrifrons*

Denis Kupsch[a,b,*], Matthias Waltert[b] & Eckhard W. Heymann[a]

[a]Behavioural Ecology and Sociobiology Unit, German Primate Centre, Göttingen, Germany

[b]Department of Conservation Biology, University of Göttingen, Germany

*Corresponding author – Email: dkupsch@gdwg.de

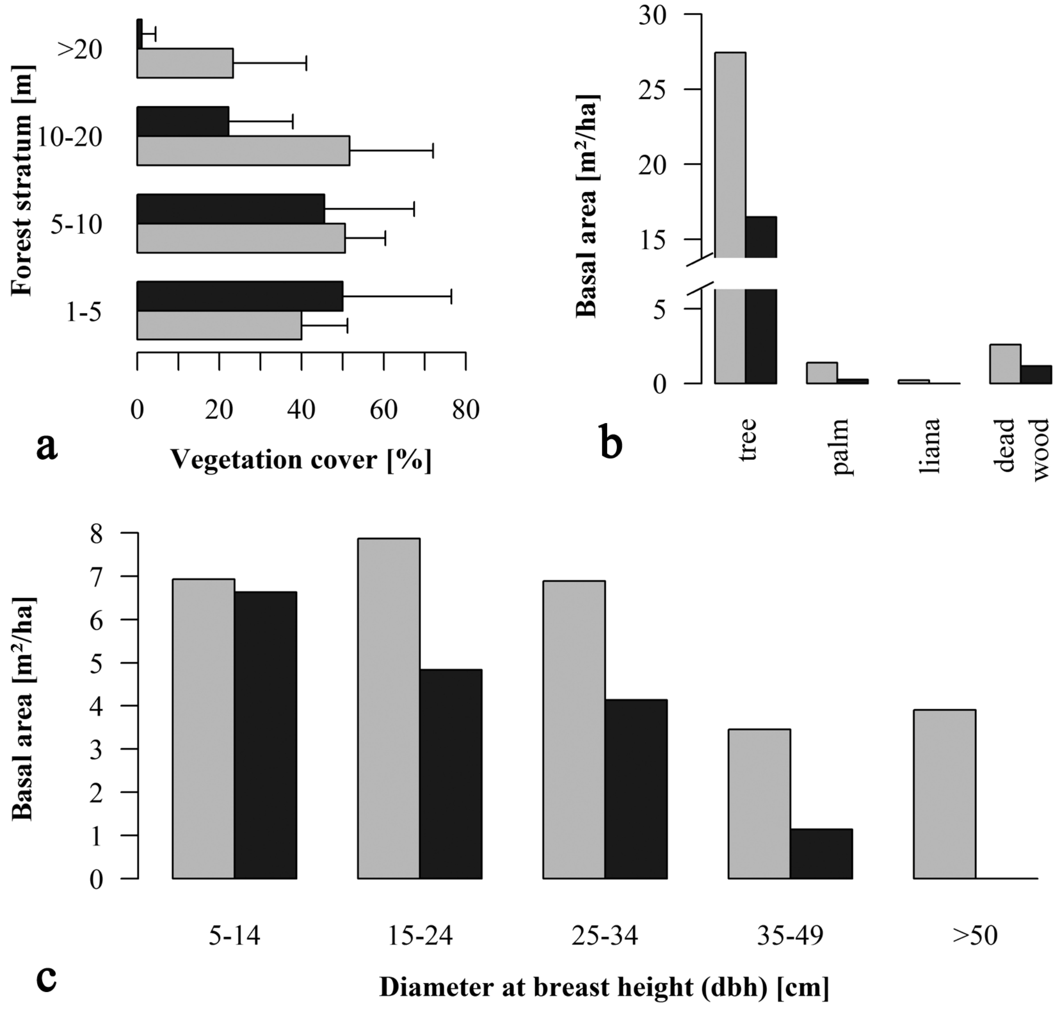

Fig. S1 Vegetation structure in primary (light grey) and secondary forest (dark grey); (a) mean vegetation cover (+SD) in different strata, (b) basal area of forest components, (c) tree composition

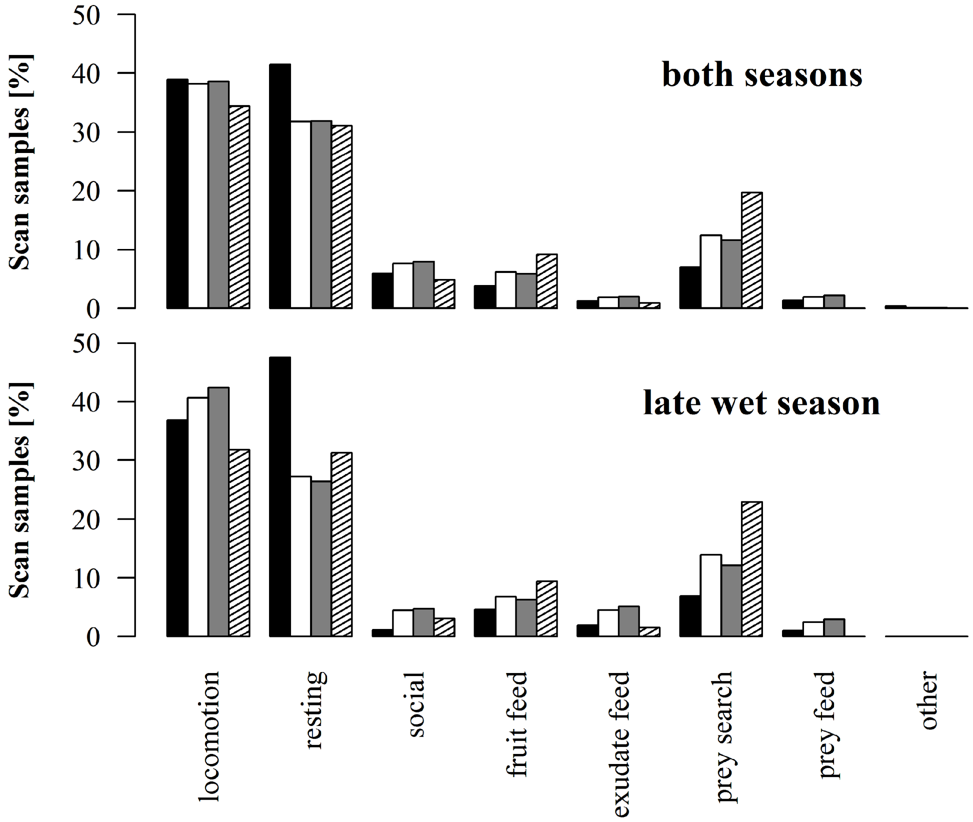

Fig. S2 Activity budgets of Group 3 (black), Group 1 (white), Group 1 in primary (grey) and in secondary forest (striped) only, across both seasons and in the late wet season

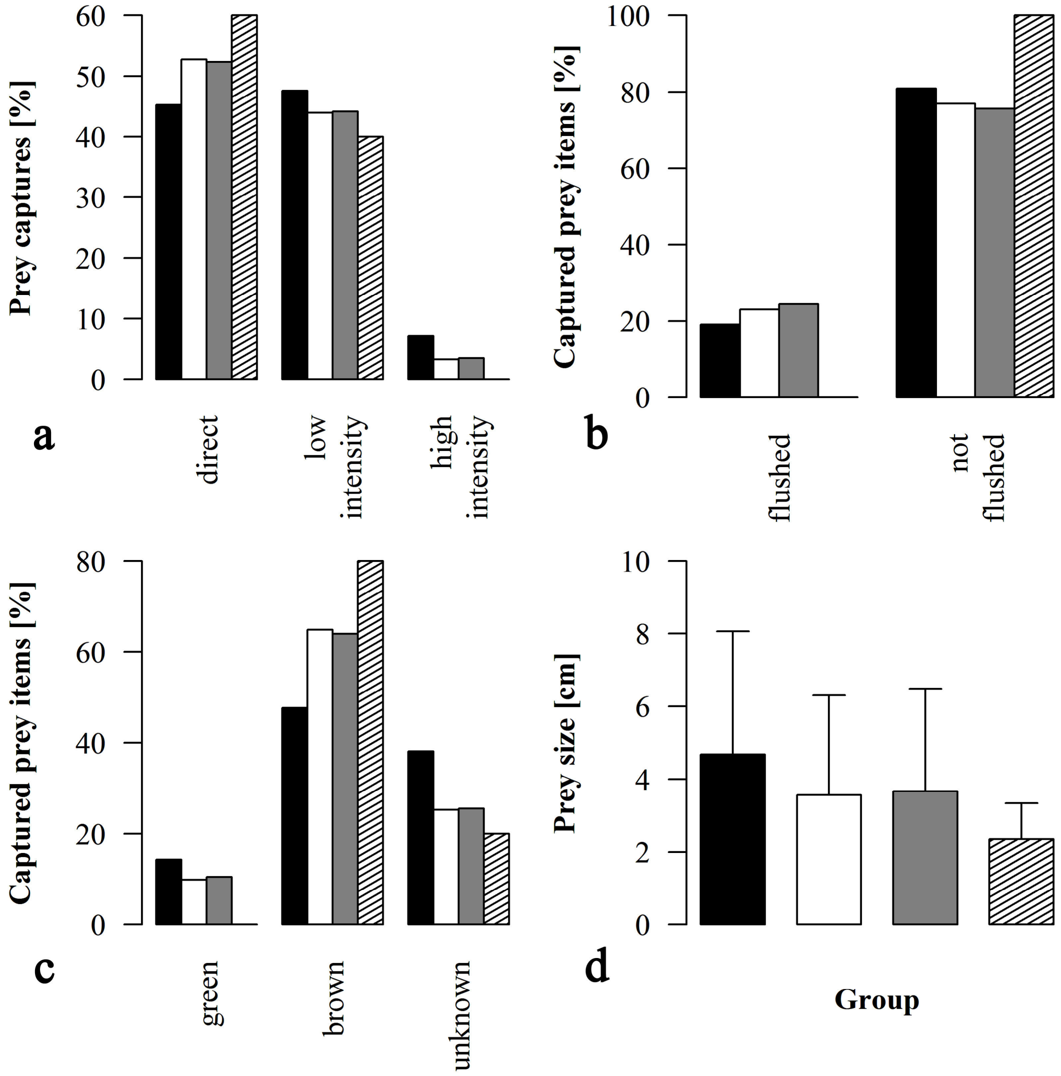

Fig. S3 Prey capture techniques: (a) type of manipulation, (b) proportion of captures resulting from prey flushing, (c) colour and (d) mean size (+SD) of captured prey items in Group 3 (black), Group 1 (white), Group 1 in primary (grey) and secondary forest (striped) only

Table S1 Frequency of captured prey taxa in Group 1 (primary and secondary forest) and 3

Taxon	Group 3	Group 1	Group 1prim	Group 1sec	Both groups
Vertebrata					
Reptilia					
Reptilia (1)		1	1		1
Reptilia (2)		1	1		1
Amphibia					
Amphibia (1)	2	2	2		4
Amphibia (2)	1				1
Amphibia (3)	1				1
Invertebrata					
Arachnida					
Araneida	8	2	17	3	28
Araneida (Eggs)	8	7	1		8
Scorpionida (1)	2	4	4		6
Scorpionida (2)	1	1	1		2
Insecta					
Blattodea		3	3		3
Mantodea		1	1		1
Coleoptera					
Coleoptera (1)	1				1
Coleoptera (2)	2				2
Phasmatodea					
Phasmatodea	1	1	1		2
Pseudophasma sp.	1	1			1
Orthoptera					
Gryllacrididae	1				1
Proscopiidae	13	8	8		21
Tettigoniidae (unidentified)	2	4	4		6
Tettigoniidae (1)	4	16	15	1	2
Tettigoniidae (2)	3	3	3		6
Tettigoniidae (3)	9	5	43	7	59
Tettigoniidae (4)	1	4	4		5
Conocephalinae					
Cophiphora gracilis	2	5	5		7
Eurymetopa obesa	2	1	1		3
Lirometopum sp.	2				2
Agraeciini		1	1		1
Pseudophyllinae					
Acanthodis longicauda	2				2
Diophanes salvifolius notatus	5	2	2		7
Macrochiton heros	1	1			1
Pterochroza ocellata	1	1			1
Schedocentrus basalis	2	6	6		8
Schedocentrus spinosus	1	2	2		3
Schedocentrus tesselatus	1				1
Typophyllum mortuifolium	1		1		1
Choeroparnops sp.	2	5	5		7
Leurophyllum sp. 1	2	1	1		3
Leurophyllum sp. 2	4	4	4		8
Leurophyllum sp. 3	1				1

Lophaspis sp.	1	1			1
Triencentrus sp.	2	1	1		3
Platyphyllini		1	1		1
Pleminiini (1)	1				1
Pleminiini (2)	1	1	1		2
Teleutiini		1	1		1
Phaneropterinae					
Anaulacomera sp.		1	1		1
Microcentrum sp.	2				2
Unidentified	37	4	39	1	77
Total	121	204	190	14	325
